# Polymorphism engineering of mefenamic acid for enhanced pharmacokinetic performance

**DOI:** 10.17179/excli2026-9258

**Published:** 2026-03-17

**Authors:** Parteek Prasher, Mousmee Sharma

**Affiliations:** 1 Department of Chemistry, University of Petroleum & Energy Studies (UPES), Dehradun, 248007, India; 2 Department of Chemistry, Uttaranchal University, Dehradun, 248007, India

## Abstract

Mefenamic acid, a BCS Class II drug, continues to face the longstanding challenges related to its suboptimal solubility and variable absorption, which necessitates frequent dosing of the drug resulting in ulcerogenicity. Guided by its polymorphic forms, the co-crystallization of mefenamic acid offers a unique advantage over the other advanced formulation strategies, including hydrotropy, nanosizing, and complexation for improving the drug bioavailability. However, the contemporary research limited only to the proof-of-concept studies fails to provide a clinical evidence or translational insights, which necessitates the rational design of synthons and engineering of the solid-state landscape of the drug for developing the co-crystallization formulation of mefenamic acid. This commentary provides critical insights into the polymorphism-driven co-crystal design of mefenamic acid aimed at filling the critical gaps in scalability and clinical translation.

## ⁯⁯

Mefenamic acid, a Biopharmaceutics Classification System (BCS) Class II drug, exhibits a poor aqueous solubility of about 0.004 % at pH 7.1, arising from its hydrophobic biphenyl structure and incomplete ionization at the physiological pH. Although pH 7.1 is above its pKa (~4.2) and promotes ionization, a substantial fraction of the drug remains in the poorly soluble unionized form, thereby requiring higher pH for a complete dissolution. Pharmacokinetic investigations have shown that mefenamic acid is rapidly absorbed after oral administration, reaching peak plasma concentrations of 10-20 mcg/mL within 2-4 hours, and exhibits a short elimination half-life of approximately 2 hours. Apparently, the steady-state plasma levels are typically achieved within 48 hours of repeated dosing, with no evidence of drug accumulation. Mefenamic acid also exhibits extensive plasma protein binding (> 90 % to albumin) and an apparent volume of distribution of about 1.06 L/kg. It undergoes primary CYP2C9-mediated biotransformation to 3-hydroxymethyl mefenamic acid, followed by further oxidation to the 3-carboxy metabolite. Both the parent compound and its metabolites undergo glucuronidation, with peak plasma levels of the hydroxy and carboxy metabolites occurring at approximately 3 hours and 6-8 hours, respectively, following a 1 g dose. Eventually, most of the drug is eliminated as its glucuronidated metabolite in urine, with a minimal excretion through faeces as an unconjugated metabolite. Hence, improving the solubility of mefenamic acid is a major challenge for the pharmaceutical formulation industry for improving the pharmacokinetic profile of the drug. Contemporary approaches encompassing hydrotropy, complexation with cyclodextrins, polymeric micellization, ionic liquids and deep eutectic solvents, salt-formation, and co-crystallization have been reported to improve the solubility constrain of mefenamic acid. In addition, advanced formulation technologies, including vesicle-based drug delivery systems, nanosuspensions, self-emulsifying drug delivery systems, and hot-melt extrusion technology have been reported to improve the dissolution rate of mefenamic acid. However, the scalability and clinical translation of these approaches is still under progress.

Among the contemporary methods under investigation to address the solubility challenge of mefenamic acid, co-crystallization offers a highly effective strategy since it maintains the molecular integrity and therapeutic effect of the API. Polymorphism plays a pivotal role in the co-crystallization behavior of mefenamic acid. Existence of two polymorphic forms of mefenamic acid, Form I (CSD, version 2024; refcode XYANAC) and Form II (CSD, version 2024; refcode XYANAC07) were identified by Aguiar and Zelmer (1969[1]), with each polymorph exhibiting distinct molecular conformations, intermolecular arrangements, and hydrogen-bonding patterns. Form I was observed to be thermodynamically stable at ambient conditions, and it adopted a more planar conformation to generate strong carboxylic acid dimers. This results in a tightly packed, low-solubility lattice relatively resistant to coformer-induced disruption. Whereas the twisted molecular conformation and lose packing arrangement in Form II favors lattice reorganization which is desirable for co-crystal formation. Romero et al. (1999[10]) showed that the latter displayed a higher dissolution rate and saturation solubility in dodecyl alcohol but undergoes a rapid transformation to Form I in the solution which may be attributed to a small free-energy difference of -251 cal/mol between the two polymorphs. This was further supported by the bioavailability studies which showed that both the polymorphs achieved similar plasma drug levels, thereby indicating that polymorphism in mefenamic acid poses a minimal impact on the drug dissolution and absorption.

Another polymorphic form of mefenamic acid, Form III (CSD, version 2024; refcode XYANAC03) was reported by SeethaLekshmi and Guru Row (2012[11]) which established the structural and conformational differences between the three polymorphs, thereby expanding the solid-state landscape of the molecule. Existence of Form III was further evidenced from the studies by Oparin et al. (2019[9]; 2023[8]) by investigating the conformational behavior of mefenamic acid in its saturated solution under supercritical CO_2_. This study also highlighted the role of thermodynamic parameters of the supercritical CO_2_ solution to understand the stability relationship and interconversion pathways among the different polymorphic forms of mefenamic acid, which assists in selectively obtaining a desired polymorph with high purity. Recently, insights into the stability of the three polymorphic forms of mefenamic acid via quantum chemical and crystallographic studies were reported by Shishkina et al. (2022[12]) which showed that the polymeric forms of the drug followed a stability order of I > II > III. In all the polymorphic forms, a centrosymmetric dimer was formed by the adjacent -COOH units of mefenamic acid where the hydrogen bonding served as a primary mode of interaction. This however did not determine the nature of packing in the crystal lattice, as the intermolecular interaction energy analysis showed that polymorphic Form I and II adopt a columnar-layered packing, whereas the Form 3 exhibited a purely columnar structure.

Insights on the polymeric behavior and lattice stability of the polymorphic forms of mefenamic acid further prompted the investigation on the effect of external processes on its crystallization behavior. Sono-crystallization studies by Mudalip et al. (2020[5]) investigated the influence of process-intensification methods on the crystallization behavior of mefenamic acid. The effects of ultrasonic power (153.3-766.7 W) and sonication time (5-30 min) during cooling crystallization of mefenamic acid in ethanol were systematically investigated. Their FTIR and PXRD analyses showed that ultrasound did not induce polymorphic transformation, with all crystallized samples consistently corresponding to Form I, evidenced by the characteristic N-H stretching band at 3313 cm^-1^ and PXRD reflections at 6.3 °, 21.3 °, and 26.3 ° (2θ). However, ultrasound had a profound impact on crystal size distribution and morphology as increasing ultrasonic power and longer sonication times produced progressively narrower CSDs and converted the typical needle-like crystals into smaller plate-like particles, attributed to cavitation-induced nucleation enhancement, crystal breakage, and disruption of crystal aggregation. Taken together, these studies show that polymorphism plays a central role in determining the solid-state behavior and co-crystallization of mefenamic acid, highlighting the importance of carefully controlling polymorphic forms to achieve consistent and rational cocrystal design.

In a first ever report, Vaksler et al. (2021[13]) synthesized single crystals of the mefenamic acid-nicotinamide (MFA:NA) co-crystal using supercritical carbon dioxide (scCO_2_), highlighting a green, solvent-free alternative to the traditional co-crystallization techniques. By systematically varying temperature, component ratio, and processing time, the authors show that co-crystallization is unsuccessful at 40 °C but proceeds efficiently at 90 °C, where a distinct powder co-crystal phase appears with characteristic new PXRD peaks, particularly when MFA:NA is present in a 1:2 molar ratio, yielding ~90 % co-crystal. DRIFTS analysis confirms co-crystal formation through shifts in nicotinamide vibrational bands, indicating new hydrogen-bonding interactions, while MFA vibrations remain largely unchanged. This study also highlighted the effectiveness of high-pressure, high-temperature supercritical CO_2_ processing in converting the entire solid to MFA-NA co-crystal (90 % conversion) as compared to the rapid expansion of supercritical solutions (RESS) method (20 % conversion). The MFA-NA co-crystal obtained after prolonged processing in supercritical CO_2_ crystallizes in a 1:2 ratio of mefenamic acid to nicotinamide. Single-crystal X-ray diffraction shows that it adopts a triclinic P-1 structure, where each MFA molecule is linked to two NA molecules through a combination of O-H···O and N-H···O hydrogen bonds. These interactions assemble into a stable hexameric unit consisting of two MFA and four NA molecules, which extends into well-ordered layers in the solid state. This hydrogen-bonded arrangement explains both the stability of the co-crystal and the successful growth of large, high-quality single crystals under prolonged supercritical CO_2_ conditions.

Application of gas antisolvent technology for the fabrication of a ternary MEF-PAR-NIC cocrystal using dense CO_2_ as an antisolvent was reported by Charoenchaitrakool et al. (2022[2]). This study demonstrated how simultaneous modulation of supersaturation, coformer stoichiometry, and precipitation temperature can be leveraged to generate novel crystalline phases with markedly superior dissolution behavior. Using a Box-Behnken design, the authors established that coformer-to-drug molar ratio and MEF saturation were the most influential variables affecting t_63.2_, and identified optimized GAS conditions (25 °C, NIC:PAR:MEF = 4.78:4.78:1, 89.74 % saturation) that yielded cocrystals with a t_63.2_ of only ~4.2 min. This observation represents a 16.4-fold improvement over unprocessed MEF. Comprehensive DSC, FT-IR, and PXRD analyses confirmed the formation of a new ternary crystalline phase distinct from the corresponding binary MEF-NIC and MEF-PAR systems, while SEM revealed characteristic cotton-ball or needle-like morphologies associated with high nucleation rates under dense-gas conditions. The study also showed that adjusting the NIC:PAR ratio enables control over the MEF:PAR mass ratio, allowing the co-crystals to align with commercial fixed-dose combinations. Overall, the work highlights that GAS-based crystallization provides a green and effective approach to produce structurally well-defined MEF multicomponent crystals with improved dissolution and promising manufacturing potential.

Nugrahani et al. (2021[7]) later showed that crystal-engineering strategies, specifically the formation of sodium mefenamate-nicotinamide multicomponent crystals, can substantially improve the solubility and dissolution of mefenamic acid. In their study, two new multicomponent crystal forms, mefenamic acid-sodium mefenamate-nicotinamide hemihydrate (SMN-HH) and monohydrate (SMN-MH) were prepared by reacting sodium mefenamate with nicotinamide in a 1:1 stoichiometric ratio to produce stable crystalline salts or cocrystal hydrates. The crystal structure of these hydrates consisted of extended hydrogen-bonded and ionic 3D networks which exposes the hydrophilic surfaces, thereby enhancing the wettability and generating channels for facilitating water penetration. This results in a rapid disintegration of the crystals as compared to the dense hydrophobic packing of pure mefenamic acid. Reportedly, both the SMN-HH and SMN-MH enhanced the solubility of sodium mefenamate at acidic pH (pH 1.2) by 4- and 3-fold, respectively. The improvement in solubility was lower in aqueous solution and buffer media, while a minimal increase was observed in basic media (pH 9.0). Similarly, both SMN-HH and SMN-MH increased the intrinsic dissolution of sodium mefenamate to 10-fold and 8-fold, respectively in acidic media (pH = 1.2). Overall, this study demonstrated that co-crystal hydrates formed through precise supramolecular design can dramatically enhance the solubility and dissolution of mefenamic acid, with SMN-HH emerging as the most promising pharmaceutical candidate.

Seminal work by Hidayat et al. (2023[4]) led to the development of MFA-nicotinamide nano-cocrystal using a sonochemical approach, reducing particle size to ~662 nm and lowering crystallinity, which together weakened lattice strength and increased surface area. The mefenamic acid-nicotinamide (MFA-NCT) nano-cocrystal reported in this study was developed from the conventional 1:2 MFA-NCT cocrystal through solvent evaporation followed by reducing its particle size using a sonochemical technique in the presence of sodium lauryl sulphate as a stabilizer. Structural characterization (PXRD, DSC, FT-IR) confirmed that both the cocrystal and nano-cocrystal possessed reduced crystallinity and altered hydrogen-bonding patterns relative to pure MFA, which is associated with lower lattice energy and easier solvent penetration. Morphological analysis showed that the cocrystal formed thin rod-shaped particles, whereas the nano-cocrystal produced submicron, flake-like particles averaging 662 ± 103 nm with a stable negative zeta potential of -42 mV, both favoring improved dispersibility. These structural changes corresponded to a marked increase in solubility: pure MFA had a solubility of 108 µg/mL, the MFA-NCT cocrystal increased it 6.7-fold, and the nano-cocrystal reached 1677 µg/mL, a 15.5-fold enhancement. A similar trend was observed in dissolution performance, with the nano-cocrystal achieving 73.72 % dissolution efficiency within 60 minutes, compared with 54.28 % for the cocrystal and 46.43 % for pure MFA. The improvement results from a synergistic effect in which cocrystal formation weakens the lattice to allow easier solvent penetration, while the nanosize increases surface area and reduces the diffusion boundary layer to accelerate dissolution. Together, these modifications show that combining nanotechnology with cocrystal engineering provides the greatest enhancement in mefenamic acid solubility and dissolution among all evaluated approaches.

George et al. (2020[3]) extended the earlier findings by showing that mefenamic acid can also form a drug-drug cocrystal with gefitinib, where altered hydrogen-bonding and tighter packing again modify solubility behavior, consistent with the structural principles highlighted by Vaksler and Nugrahani. However, unlike the nicotinamide systems, the GFN-MFA cocrystal exhibits only a modest increase in MFA solubility and even slows Gefitinib dissolution, underscoring how the specific partner molecule and resulting supramolecular architecture critically dictate biopharmaceutical outcomes. Design and characterization of a 1:1 drug-drug cocrystal hydrate of gefitinib (GTB) and mefenamic acid (MFN) were developed to explore synergistic solid forms for oncology treatment while simultaneously improving the physicochemical limitations of both drugs. The GTB-MFN cocrystal crystallizes in the monoclinic P2₁/c space group, containing one molecule each of GTB and MFN along with one water molecule, which plays a critical role in stabilizing the lattice. The π-stacking interactions, and intramolecular hydrogen bonding between -COOH of mefenamic acid and -NH of the quinazoline ring of gefitinib results in a rigid supramolecular framework in co-crystals. These co-crystals significantly enhanced the solubility and dissolution of MFN by 3-fold as compared to its Polymorph Form 1, owing to the disruption of densely packed co-crystal.

Overall, most of the published studies remain predominantly exploratory and proof-of-concept in nature, focusing on solid-state characterization and short-term dissolution testing, while lacking comprehensive mechanistic understanding of how co-crystallization alters intermolecular energetics, molecular mobility, and solvent-mediated transformation pathways at physiological and manufacturing-relevant conditions. Furthermore, there is a scarcity of systematic 'structure-property relationships' as co-former selection still relies largely on empirical screening rather than predictive supramolecular design frameworks incorporating computational modelling, machine learning, or crystal landscape mapping. Another major gap is the limited study of scale-up feasibility and manufacturability as most co-crystal systems are produced through laboratory-scale methods such as solvent evaporation or small-batch mechanochemistry, with very few reports evaluating robustness under continuous processing, hot-melt extrusion, spray-crystallization, or industrial milling. Likewise, there is insufficient information regarding the long-term physical and chemical stability of mefenamic acid co-crystals under ICH storage conditions, particularly in relation to polymorphic conversion, hydrate/solvate formation, mechanical stress during tableting, and humidity-triggered dissociation. Finally, although several co-crystals demonstrate improved solubility and dissolution *in vitro*, an absence of *in vivo* pharmacokinetic and pharmacodynamic evidence creates a sizeable challenge in their clinical translation for improved bioavailability, safety, or therapeutic performance.

In conclusion, co-crystallization of mefenamic acid and its clinical outcomes continue to be explored. Advanced approaches such as high-pressure crystallization studies show multiple polymorphs of MA, which influences its co-crystallization kinetics, crystallization pathway, and final solid form (Nakapraves et al. 2022[6]). Secondly, the stability of co-crystals presents a major challenge, as in the case of MA-nicotinamide systems, where a salt-cocrystal study of sodium mefenamate-nicotinamide reported that different hydrate forms interconverted under ambient conditions. Additionally, the crystallization method and processing conditions profoundly affect the outcome, and scaling such processes is challenging. This was evident in the study reporting a gas-antisolvent process for MA-paracetamol which required careful optimization to produce the desired co-crystal. Further, even when co-crystals are obtained, demonstrating improvement in their properties under real formulation/manufacturing/storage conditions remains challenging, as co-crystals may show improved dissolution in controlled lab experiments, but the influence of downstream processes, humidity, tableting stress or polymorphic conversion is often under-explored. Hence, although the co-crystallization of mefenamic acid holds promise for improving its performance, the combination of polymorphic risk, process sensitivity, stability concerns and translation into scalable manufacturing present a significant barrier to robust development.

## Declaration

### Conflict of interest

The authors declare no conflict of interest.

### Artificial Intelligence (AI) - assisted technology

The authors declare that they have not used artificial intelligence for the writing of the manuscript.

## References

[1] Aguiar AJ, Zelmer JE (1969). Dissolution Behavior of Polymorphs of Chloramphenicol Palmitate and Mefenamic Acid. J Pharm Sci.

[2] Charoenchaitrakool M, Roubroum T, Sudsakorn K (2022). Processing of a novel mefenamic acid-paracetamol-nicotinamide cocrystal using gas anti-solvent process. J CO2 Utilization.

[3] George CP, Thorat SH, Shaligram PS, Suresha PR, Gonnade RG (2020). Drug-Drug Cocrystals of Anticancer Drugs Erlotinib-Furosemide and Gefitinib-Mefenamic acid for Alternative Multi-Drug Treatment. Cryst Eng Comm.

[4] Hidayat AF, Sofiandini L, Darusman F (2023). Development and Characterization of Mefenamic Acid-Nicotinamide Nano-Cocrystal. SSNR 4556716.

[5] Mudalip SKA, Sezali NA, Abu Bakar MR (2020). Effect of ultrasonic waves on polymorphism and crystal size distributions of mefenamic acid. IOP Conf Ser: Mater Sci Eng.

[6] Nakapraves S, Warzecha M, Mustoe CL, Srirambhatla V, Florence AJ (2022). Prediction of Mefenamic Acid Crystal Shape by Random Forest Classification. Pharm Res.

[7] Nugrahani I, Fisandra F, Horikawa A, Uekusa H (2021). New Sodium Mefenamate - Nicotinamide Multicomponent Crystal Development to Modulate Solubility and Dissolution: Preparation, Structural, and Performance Study. J Pharm Sci.

[8] Oparin RD, Krestyaninov MA, Ivlev DV, Kiselev MG (2023). Molecular Mechanism of Conformational Crossover of Mefenamic Acid Molecules in scCO2. Materials (Basel).

[9] Oparin RD, Vaksler YA, Krestyaninov MA, Idrissi A, Shishkina SV, Kiselev MG (2019). Polymorphism and conformations of mefenamic acid in supercritical carbon dioxide. J Supercritical Fluid.

[10] Romero S, Escalera B, Bustamante P (1999). Solubility behavior of polymorphs I and II of mefenamic acid in solvent mixtures. Int J Pharm.

[11] SeethaLekshmi S, Guru Row TN (2012). Conformational Polymorphism in a Non-steroidal Anti-inflammatory Drug, Mefenamic Acid. Cryst Growth Des.

[12] Shishkina SV, Vaksler YA, Konovalova IS, Dyakonenko VV, Varchenko VV (2022). Quantum Chemical Study on Mefenamic Acid Polymorphic Forms. ACS Omega.

[13] Vaksler YA, Benedis D, Dyshin AA, Oparin RD, Correia NT, Capet F (2021). Spectroscopic characterization of single co-crystal of mefenamic acid and nicotinamide using supercritical CO2. J Mol Liq.

